# Tracing the legacy of the early Hainan Islanders - a perspective from mitochondrial DNA

**DOI:** 10.1186/1471-2148-11-46

**Published:** 2011-02-15

**Authors:** Min-Sheng Peng, Jun-Dong He, Hai-Xin Liu, Ya-Ping Zhang

**Affiliations:** 1 State Key Laboratory of Genetic Resources and Evolution, Kunming Institute of Zoology, Chinese Academy of Sciences, Kunming, P.R. China; 2 School of Life Science, University of Science and Technology of China, Hefei, P.R. China; 3 KIZ/CUHK Joint Laboratory of Bioresources and Molecular Research in Common Diseases, Kunming, P.R. China; 4 Laboratory for Conservation and Utilization of Bio-resources, Yunnan University, Kunming, P.R. China; 5 Graduate School of the Chinese Academy of Sciences, Beijing, P.R. China; 6 Current address: Program in Neuroscience, SUNY at Stony Brook, Stony Brook, New York, USA

## Abstract

**Background:**

Hainan Island is located around the conjunction of East Asia and Southeast Asia, and during the Last Glacial Maximum (LGM) was connected with the mainland. This provided an opportunity for the colonization of Hainan Island by modern human in the Upper Pleistocene. Whether the ancient dispersal left any footprints in the contemporary gene pool of Hainan islanders is debatable.

**Results:**

We collected samples from 285 Li individuals and analyzed mitochondrial DNA (mtDNA) variations of hypervariable sequence I and II (HVS-I and II), as well as partial coding regions. By incorporating previously reported data, the phylogeny of Hainan islanders was reconstructed. We found that Hainan islanders showed a close relationship with the populations in mainland southern China, especially from Guangxi. Haplotype sharing analyses suggested that the recent gene flow from the mainland might play important roles in shaping the maternal pool of Hainan islanders. More importantly, haplogroups M12, M7e, and M7c1* might represent the genetic relics of the ancient population that populated this region; thus, 14 representative complete mtDNA genomes were further sequenced.

**Conclusions:**

The detailed phylogeographic analyses of haplogroups M12, M7e, and M7c1* indicated that the early peopling of Hainan Island by modern human could be traced back to the early Holocene and/or even the late Upper Pleistocene, around 7 - 27 kya. These results correspond to both Y-chromosome and archaeological studies.

## Background

**Figure 1:**
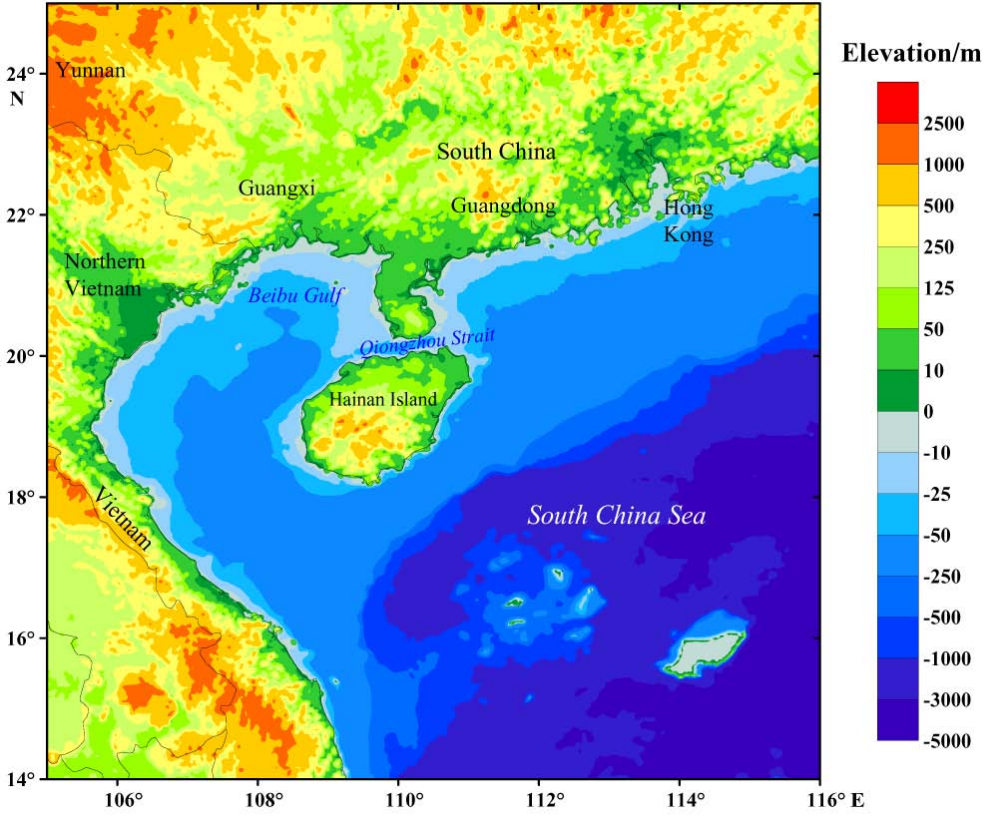
**Map of Hainan Island and its surrounding regions, showing elevation relative to modern sea level**. Map outline was kindly provided by YT. Yao, CAS Key Laboratory of Marginal Sea Geology, South China Sea Institute of Oceanology, Guangzhou [3].

Hainan Island, the second largest island of China, is located in the Beibu Bay (Gulf of Tonkin) and separated from Guangdong's Leizhou Peninsula to the north by the Qiongzhou Strait (Figure 1). During the Last Glacial Maximum (LGM), around 19 - 26.5 kya (thousand years ago) [1], Hainan Island was connected with mainland southern China and northern Vietnam, as the sea level was around 80 - 100 m below present day [2,3]. Thus, Hainan Island might lay on one of the modern human northward migration routes from Southeast Asia to East Asia and it is likely that Hainan islanders may maintain certain ancient footprints of these dispersals [4]. This scenario has been supported by some recent archaeological evidence, which suggested that Hainan Island might have been colonized by human in the Upper Pleistocene or the Upper Paleolithic period [5-8].

Due to the post-glacial sea level rising and the formation of the Qiongzhou Strait, Hainan Island has been isolated from the mainland for at least 6 thousand years [3,9]. Presently, Hainan Island is home to people with many different languages and/or cultures. Compared with other ethnic/linguistic groups (e.g. Lingao, Han, and Hmong) - the recent immigrants from mainland southern China [10], Li (Hlai) people were suggested to be the earliest settlers, having arrived in Hainan Island at least 3 kya [11]. In terms of linguistic analyses, the Hlai language, used by the Li people was suggested to split from other languages within the Tai-Kadai (Daic) family ~ 3 - 4 kya [12]. Meanwhile, some current Li populations still maintain some ancient cultures of the Neolithic, e.g. bark cloth and original ceramic making [10]. Therefore, colonization of Hainan Island by the ancestors of the Li people can be at least traced back to the Neolithic period (~ 2 - 6 kya) [11,13]. However, whether the ancestor of the modern human had settled in this region in the Upper Pleistocene and contributed to the gene pool of modern Li populations in Hainan Island is unclear.

To depict the prehistoric peopling events in this region, human genetic approaches based on the uniparental genetic systems - mitochondrial DNA (mtDNA) and the nonrecombining region of the Y chromosome (NRY) - have been widely adopted [14]. By analyzing the dominant NRY haplogroups (paragroups) O1a* and O2a* in Hainan aborigines (five Li populations and one Cun population), Li et al. suggested that Hainan aborigines had been isolated at the entrance to East Asia for ~ 20 thousand years [4]. However, because of the relatively poor resolution of phylogeny based on limited numbers of Y-SNPs, the candidate founders of the ancient dispersal were still ambiguous. Moreover, in their later work about O1a*, wide connections among the populations around the Beibu Bay (i.e. Guangxi and Hainan) and other populations from southern China and Southeast Asia were observed [15]. This implies that the effect of some recent gene flows between Hainan islanders and the populations in the mainland could not be ignored.

In this study, we adopted mtDNA analyses to trace the ancient peopling of Hainan Island in a maternal perspective, because: 1) the phylogeny of mtDNA in context of East Asian and Southeast Asian has been improved thanks to large scale complete mtDNA genome sequencing [16-28]; 2) mtDNA data of many ethnic/linguistic groups in the neighboring regions of Hainan Island (i.e. southern China [29-36] and northern Vietnam [23,37]) have been reported. Given that the maternal structures of the Li populations were poorly characterized in previous work [30], we collected new samples from 285 Li individuals. With comprehensive phylogeographic analyses based on complete mtDNA genomes sequencing, we identified some potential candidate markers for the early peopling of Hainan Island, which could be traced back to ~ 7 - 27 kya.

## Results

### The phylogeny of mtDNA in Hainan Island

**Figure 2:**
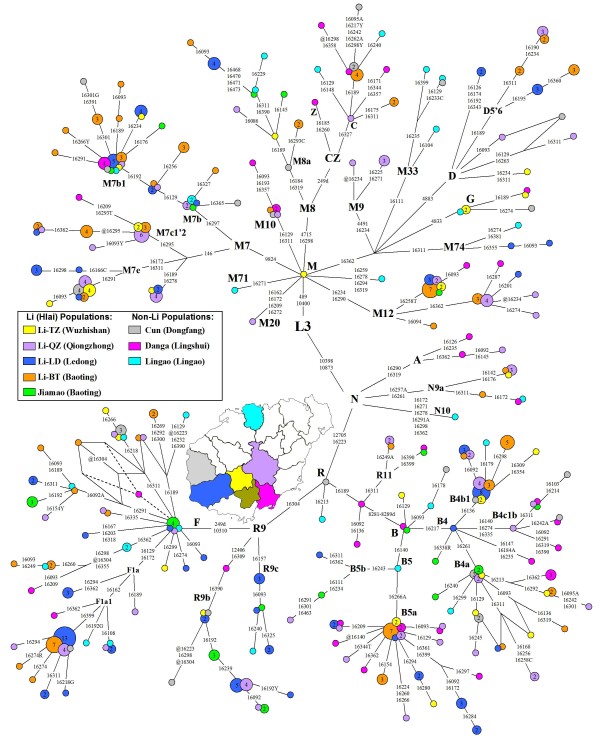
**Tree drawn from a median-joining network of 180 mtDNA haplotypes observed in Hainan Island**. mtDNA motifs of HVS-I (16080-16488) combined with HVS-II and/or certain coding region sites were considered to improve the resolution of the tree which was constructed manually and checked by using the Network 4.510. The circles represent mtDNA sequence types, shaded according to population with an area proportional to their absolute frequency. The geographic sources of populations were also noted. These are transitions while suffixes A, C, G and T refer to transversions, "Y" specifies heteroplasmic status C/T at the site, and "@" means a reverse mutation. Seven haplotypes were determined as the existing of heteroplasmic sites.

Hypervariable sequence (HVS) analysis and partial coding region testing indicated that all mtDNA lineages of the 285 Li individuals were unambiguously assigned into the previously defined haplogroups in East and Southeast Asians (see Additional file 1). The predominant haplogroups in southern China and Southeast Asia: haplogroups B, F, and M7 together account for ~ 69%, 71%, and 63% of the maternal gene pools of the populations Li-BT, Li-LD, and Li-QZ, respectively (Table 1). The prevailing haplogroups in northern China, such as haplogroups A, D4, G, and Z, were rare or even absent in the three Li populations. Meanwhile, the previously reported 162 sequences from five populations (Li-TZ, Jiamao, Cun, Danga, and Lingao) in Hainan Island [30] were re-evaluated and incorporated into further analyses. The skeleton of the resulting phylogeny of 447 individuals from Hainan Island was constructed (Figure 2). The mtDNA haplogroups profiles of the Hainan islanders were similar to the patterns observed in the populations from the mainland southern China and northern Vietnam (Table 1). However, haplogroups M12 and M7e had higher frequencies (~ 6.3% and ~ 4.5%, respectively) in the Hainan islanders than those of the populations in the mainland, whose average were less than 1%.

**Table 1:** mtDNA haplogroup frequencies in Hainan Island, Taiwan, mainland southern China, and Vietnam

	Hainan								Hainan	Taiwan	Guangxi	Guangdong	Hong Kong	Yunnan-SE	N-Vietnam	M-Vietnam	Mainland
	Li-BT	Li-LD	Li-QZ	Li-TZ	Jiamao	Cun	Lingao	Danga	Total	Total	Total	Total	Total	Total	Total	Total	Total
Haplogroups	n = 99	n = 100	n = 86	n = 34	n = 27	n = 30	n = 31	n = 40	n = 447	n = 640	n = 1111	n = 546	n = 337	n = 158	n = 326	n = 66	n = 2584
A			1.16					5.00	0.67		1.71	2.56	3.98	0.63	0.92		2.01
B*				2.94	3.70	3.33	6.45	2.50	1.34		1.08	0.55	0.80	1.90	0.92	4.55	1.04
B4*		1.00			3.70				0.45		1.71	2.20	1.86	1.27	2.76	1.52	1.93
B4a	2.02	1.00	5.81	8.82	11.11	13.33	3.23	7.50	4.92	15.63	6.03	5.13	2.65	4.43	3.37	1.52	4.80
B4b1	9.09	11.00	6.98	8.82		3.33	3.23		6.94	7.34	3.24	3.48	2.39	2.53	0.61		2.71
B4c1b			2.33		3.70	6.67		7.50	1.79	3.91	0.72	2.75	2.39		2.15	1.52	1.55
B4c2								2.50	0.22		0.36	0.55		3.16	1.23	4.55	0.74
B4g	2.02		1.16	2.94					0.89		2.16	1.47	0.80	0.63	3.07		1.78
B5a	10.10	10.00	8.14	8.82				17.50	8.28	5.16	7.92	4.40	5.57	5.70	11.66	3.03	7.04
B5b		1.00	1.16		3.70				0.67		0.72	1.65			0.61	1.52	0.77
C	6.06		1.16			10.00	6.45	7.50	3.36		4.32	2.38	3.71	6.96	3.68	3.03	3.87
D4		2.00	2.33	2.94		3.33			1.34	1.25	4.77	10.62	11.41	6.96	2.76	1.52	6.77
D5'6	7.07	3.00	3.49						2.91	4.06	1.89	5.13	6.37	2.53	2.15	1.52	3.29
E										12.03		0.18					0.04
F*				2.94			6.45		0.67		0.63	0.18					0.31
F1*	3.03		1.16				3.23		1.12		2.07	3.48	1.59	4.43	2.15	1.52	2.44
F1a*		3.00	1.16						0.89	2.19	4.05	4.95	3.71	3.16	7.36	9.09	4.68
F1a1*	9.09	16.00	5.81			3.33	3.23	2.50	7.38	3.28	3.42	4.03	5.04	4.43	4.29	6.06	4.02
F1a1a		2.00	1.16				3.23		0.89		3.51	3.11	2.92	2.53	5.83	9.09	3.72
F2	4.04	6.00	3.49		25.93		6.45	2.50	5.15	0.16	1.62	3.30	3.45	0.63	0.61		2.01
F3	3.03						3.23	2.50	1.12	8.44	3.96	3.48	2.12	5.70	0.92		3.21
F4			1.16	5.88		10.00	3.23		1.57	11.25	0.72	0.92	0.53		0.31		0.62
G				8.82		3.33	3.23	2.50	1.34	0.16	2.07	2.38	1.06	7.59	0.92	3.03	2.21
M*				2.94			3.23		0.45		2.88	0.92	3.18	3.16	3.37	7.58	2.71
M10	1.01		1.16			3.33		7.50	1.34	0.63	1.08	2.01	1.86	0.63	0.92		1.32
M12	11.11	4.00	9.30	5.88	3.70			5.00	6.26	0.31	0.72	0.73		3.16	1.53	1.52	0.89
M20			1.16						0.22		0.09	0.37			0.61		0.19
M33						3.33	9.68		0.89		1.17	0.92	1.33		0.61		0.97
M71							3.23		0.22		0.54	0.37	0.27	1.27	0.92	3.03	0.62
M74		2.00					3.23		0.67		0.54	0.18	0.80	0.63		3.03	0.50
M7b*	5.05	3.00	3.49		3.70	3.33	6.45		3.36	11.56	6.75	4.95	5.84	9.49	5.83	6.06	6.27
M7b1	10.10	9.00	1.16	5.88	7.41	6.67	3.23	10.00	6.94	0.78	9.09	3.66	6.63	5.70	6.75	6.06	7.00
M7c1'2	3.03		6.98	5.88				2.50	2.68	6.56	1.89	2.56	1.33		2.76	1.52	1.93
M7c3	6.06	1.00	2.33						2.01		1.44	1.83	1.59		1.23		1.39
M7c*		2.00	4.65	2.94					1.57		0.09						0.04
M7e	2.02	5.00	4.65	14.71		13.33			4.47		0.18		0.53		0.61		0.23
M8a	3.03	4.00	1.16	2.94	7.41	3.33	6.45		3.13		1.08	2.38	1.06		0.92	1.52	1.28
M9a'b			4.65						0.89		1.53	0.73	1.59	1.27	1.53	1.52	1.35
N*											0.99	0.18		0.63	1.23		0.66
N10							3.23		0.22		0.09	0.37	0.53		0.61		0.27
N9a	2.02		3.49	2.94			3.23	2.50	1.79	1.56	3.42	3.85	2.12		3.37	1.52	3.06
R*						3.33	3.23	5.00	0.89		1.08	0.18	1.59	1.27	0.92		0.93
R11	1.01		2.33		3.70			2.50	1.12		1.08	0.55	0.80				0.70
R9*											0.27			1.27	0.92	3.03	0.39
R9b		8.00	5.81	2.94	18.52	6.67		2.50	4.92		3.96	1.47	2.12	3.16	3.37	7.58	3.13
R9c		6.00			3.70		3.23		1.79	2.34	0.63	0.73	2.12	1.27	1.53	3.03	1.08
Y										1.41	0.36	0.37	0.53				0.31
Z								2.50	0.22		0.36	1.83	1.86	1.90	2.15		1.20
**Haplotype** **Diversities**	**0.976**	**0.966**	**0.982**	**0.980**	**0.960**	**0.972**	**0.996**	**0.986**	**0.987**	**0.966**	**0.989**	**0.996**	**0.994**	**0.991**	**0.992**	**0.988**	**--**

### Comparison of the Hainan islanders with other populations in the mainland

**Figure 3:**
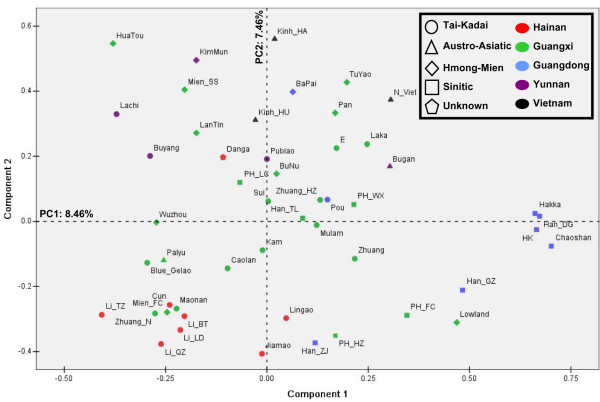
**Principle components analysis (PCA) of populations in southern China and Vietnam**. The detailed information of 50 populations employed and their haplogroup profiles was indicated in Additional file 2 and 3.

**Figure 4:**
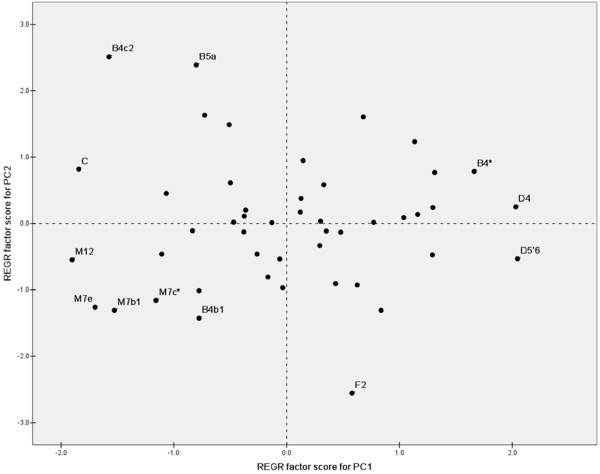
**Plot of the haplogroup contribution of the first and second PC**. The contribution of each haplogroup was calculated as the factor scores for PC1 and PC2 with regression method (REGR) in SPSS13.0 software.

To compare the Hainan Islanders with other populations in the mainland (see Additional file 2), the principal components (PC) analysis based on haplogroup frequencies (see Additional file 3) was performed (Figure 3). In the first PC, some Sinitic populations (Han-DG, HK, Hakka, and Chaoshan) clustered in one pole. In the other pole, except Lingao and Danga, most Hainan islanders were clustered with some populations from Guangxi, which were distinguished from the other populations in the mainland by the second PC. The genetic difference between Hainan islanders and populations from the mainland was statistically significant (*p *< 0.001, Analysis of molecular variance, AMOVA), whereas the difference between the Li populations (Li-BT, Li-LD, Li-QZ, Li-TZ, and Jiamao) and non-Li populations (Cun, Danga, and Lingao) was not (*p *= 0.147 ± 0.010, AMOVA). Then, we used the regression method to estimate the contribution of each haplogroup to the PCs [38]. The haplogroups M12, M7e, M7b1, M7c*, and B4b1 were found to contribute most to the pole consisting of Hainan islanders and the populations from Guangxi (Figure 4).

### Dissection of mtDNA haplotypes in Hainan Island

To analyze mtDNA variation at a finer level, we dissected the haplotype information mainly based on HVS-I segment 16080 - 16488 (Figure 2). In general, the Hainan Island populations showed fairly high haplotype diversities compared with Taiwan aborigines or other populations from the mainland (Table 1). Some haplotypes were shared by the Li and non-Li populations within Hainan Island. In total of 178 haplotypes (16090 - 16365) observed in Hainan islanders, 80 types could be found the identical counterparts in the mainland (see Additional file 4). In addition, the median-joining networks of the most frequent haplogroups (B4b1, B5a, F1a1, R9b, and M7b1) were unable to identify candidate founder types which were suitable to date the related peopling of Hainan Island (see Additional file 5).

For the rest of the three haplogroups (i.e. M12, M7e, and M7c*) contributing most to the pole of Hainan islanders in PCA (Figure 4), some interesting patterns were observed. With HVS-I motifs as 16223-16234-16258T-16290, 16189-16223-16278 and 16166C-16172-16223-16311, three lineages assigned within haplogroups M12, M7c* and M7e, respectively, were restricted in Hainan Island. Meanwhile, the lineages of 16223-16234-16258T-16290 and 16166C-16172-16223-16311 underwent certain sub-differentiation to generate the derived lineages (Figure 2). Moreover, haplogroups M12, M7c* and M7e were more concentrated in the Li populations than those in other non-Li populations (Table 1). Hence, this implies that the three haplogroups might be useful to trace the early peopling of Hainan Island.

### Candidate markers for the early peopling of Hainan Island

**Figure 5:**
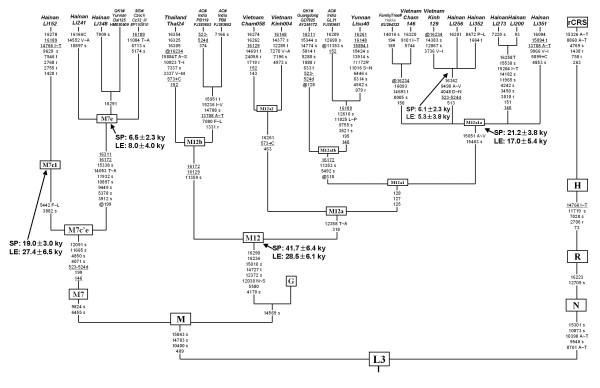
**Reconstructed phylogenetic tree of 21 complete mtDNA genome sequences from haplogroups M12 and M7c'e**. The six reported sequences were taken from the literature and were further labeled by the symbols MD [18], AC [39], QK1 [20], and QK2 [28] followed by "#", the geographic locations, and the sample codes or the access numbers in GenBank. One sequence (Accession No. EU294322) submitted by "Family Tree DNA" was retrieved from GenBank. Haplogroup age estimates (±standard errors) are indicated at the branch roots in terms of the calibrated mutation rate with symbols as SP [58] and LE [66], respectively. Mutations are transitions at the respective nucleotide position unless otherwise specified. Letters following positions indicate transversions. Recurrent mutations are underlined. +: insertion; d: deletion; @: back-mutation. "R" specifies heteroplasmic status A/G and was also noted in italic. Amino acid replacements are specified by single-letter code; s, synonymous replacements; t, change in transfer RNA; r, change in ribosomal RNA gene.

As the available phylogeny of haplogroups M12, M7e, and M7c* has not been well depicted at a fine-grained level, we sequenced 14 complete mtDNA genomes: eleven from haplogroup M12, two from haplogroup M7e and one from haplogroup M7c* (Figure 5). The phylogeny of haplogroup M12 was much improved compared to previous work [20,39]. As the result of the earliest split, haplogroup M12b was defined by mutations 11359-16129-16172 and haplogroup M12a was determined by 318-12358. Within haplogroup M12a, haplogroup M12a2 was newly determined by two sequences from Vietnam as sharing mutations 463-16261 and C insertion at 573. Haplogroup M12a1 was defined by the sequence variation motif in HVS-II as 125-127-128 and then was further divided into two clades as M12a1a and M12a1b. All sequences from Hainan Island were clustered with the sequences from Guangdong and Vietnam into haplogroup M12a1a defined by transitions 15463-15651. The unique lineage of Hainan islanders with HVS-I motif as 16223-16234-16258T-16290 was derived directly from the root of M12a1a suggesting that the modern human colonization of Hainan Island was likely to be associated with the differentiation of M12a1a around 17 - 20 kya.

**Figure 6:**
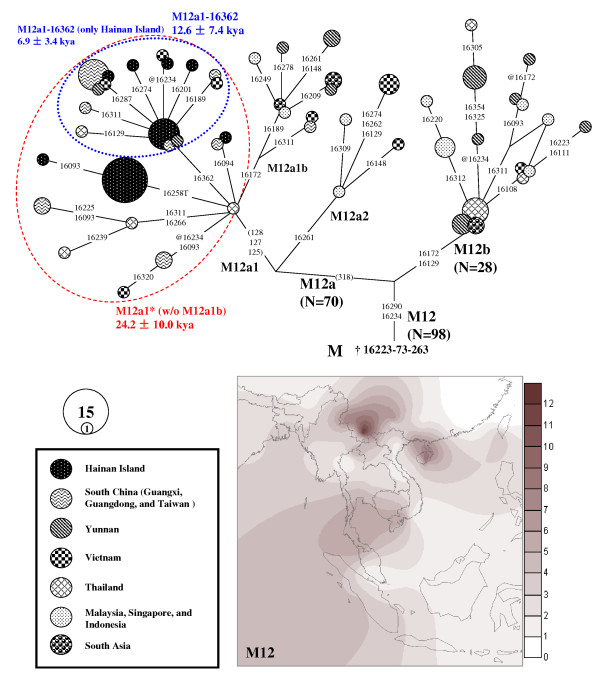
**Median-joining network of HVS-I sequences of haplogroup M12 and the spatial frequency distribution**. The circles represent mtDNA HVS-I (16090 - 16365) sequence types, shaded according to region with an area proportional to their absolute frequency which is also indicated by the number in the circle. Mutations are transitions unless the base change is explicitly indicated. Heteroplasmic positions are indicated by an "H" after the nucleotide positions. Some HVS-II sites were employed to improve the resolution and were noted in parentheses.

To characterize the phylogeographic pattern of haplogroup M12, the median-joining network was constructed with all available M12 mtDNAs (Figure 6; see Additional file 6). Haplogroup M12 was widely distributed in southern China, Southeast Asia and the eastern part of India, but relatively concentrated in Yunnan and Hainan Island (Figure 6). The network suggested haplogroup M12 was likely to originate from mainland southern China and Southeast Asia. The estimated expansion time of M12a1*, which includes the lineages from Hainan Island, was 24.2 ± 10.0 kya. The expansion of M12a1-16362 lineages in Hainan Island was estimated as 6.9 ± 3.4 kya. The results were largely in agreement with the results from the complete mtDNA genomes (Figure 5). The two ages would be considered as the potential upper (~ 24 kya) and lower (~ 7 kya) limits for the peopling of Hainan Island represented by haplogroup M12, respectively.

**Figure 7:**
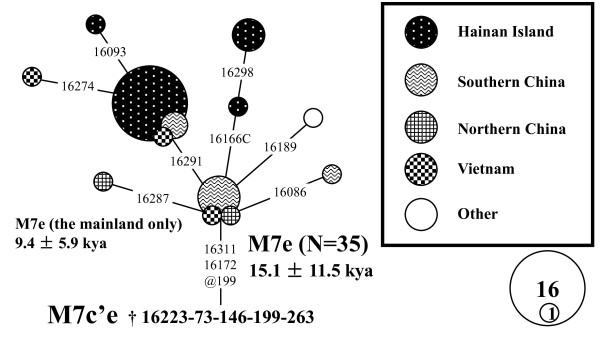
**Median-joining network of HVS-I sequences of haplogroup M7e**. The circles represent mtDNA HVS-I (16085 - 16365) sequence types, shaded according to region with an area proportional to their absolute frequency which is also indicated by the number in the circle. Mutations are transitions unless the base change is explicitly indicated. Heteroplasmic positions are indicated by an "H" after the nucleotide position.

The phylogeny of haplogroup M7e based on complete mtDNA genomes revealed that the sequence (Li241) with 16166C-16172-16223-16311 was directly derived from the root of haplogroup M7e around 6.5 - 8.0 kya (Figure 5). As the lineages with the proto haplotypes defined by HVS-I variations 16172-16223-16311 were mainly found in southern China and Vietnam (see Additional file 7), the colonization of Hainan Island represented by haplogroup M7e would be probably from this region around 15.1 ± 11.5 kya (Figure 7). However, as the network of haplogroup M7e was not in the ideally star-like structure, the time estimates with a huge standard error should be treated with caution. When we estimated the expansion time of M7e without the Hainan data, the age (9.4 ± 5.9 kya; Figure 7) seemed more compatible with the result based on mtDNA genomes (Figure 5).

For the sequences of M7c* with HVS-I motif as 16189-16223-16278, the complete mtDNA sequence (Li152) could be assigned into M7c1 but did not cluster with any known lineages of M7c1. This pattern implied that the related peopling of Hainan Island was likely to be traced back to the initial differentiation of haplogroup M7c1 as early as ~ 18 - 27 kya (Figure 5).

## Discussion

In general, the mtDNA haplogroup profiles of Hainan islanders are similar to the profiles of the populations from mainland southern China. This pattern is consistent with the previous work on NRY [4,40-43]. It suggests the Hainan islanders should have derived from mainland southern China and/or have had a common origin with the populations from this region [30]. Especially, most Hainan islanders were clustered with some populations from Guangxi (Figure 3). This pattern was also reflected by the genome-wide data: the Jiamao population in Hainan Island was clustered with the Zhuang population (i.e. the dominant minority ethnic group in Guangxi) as a branch in the tree of the "Pan-Asian" [44]. Thus, the ancestors of the Li populations were likely from Guangxi.

As the prevailing haplotype sharing has been found between the Hainan islanders and the populations from the mainland, the role of the recent gene flow from the mainland in shaping the maternal pool of Hainan islanders can not be ignored. This result is in agreement with the archaeological research, which revealed that the tight links between Hainan Island and mainland southern China existed during the Neolithic period [5,10,13]. Meanwhile, some haplotypes were shared by the Li (original aborigines) and the non-Li (recent immigrants) populations (Figure 2). It suggests that intermarriages among different populations might be common and could strengthen the effect of recent demographic events. As a result, although certain haplogroups in Hainan islanders present the star-like phylogeny in the network (e.g. B5a and M7b1, Figure 2; see Additional file 5) and the characteristics of the candidate founders [45], whether their expansion time estimates (Table 2) could be associated with the early peopling of Hainan Island is still elusive.

**Table 2:** Coalescence ages of the most frequent mtDNA haplogroups in Hainan Island

	Total					Li				
Haplogroups	Individuals	ρ	σ	T	ΔT	Individuals	ρ	σ	T	ΔT
B5a	37	1.03	0.32	19.4	5.9	30	1.07	0.35	20.1	6.6
B4b1	31	0.45	0.26	8.5	4.9	29	0.45	0.27	8.4	5.2
F1a1-16399	31	0.13	0.07	2.4	1.2	30	0.13	0.07	2.5	1.3
M7b1	31	0.45	0.22	8.5	4.2	24	0.54	0.29	10.2	5.0
M12	28	0.61	0.40	11.4	7.6	26	0.54	0.39	10.1	7.4
R9b	21	1.67	1.00	31.4	18.8	19	1.21	1.10	31.7	20.7
M7e	20	1.00	0.82	18.8	15.4	16	1.00	0.78	18.8	14.6

To trace the early peopling of Hainan Island, we paid more attention to haplogroups M12 M7c1*, and M7e, because: 1) they have relatively high frequencies in Hainan Island and are relatively concentrated in the Li populations; 2) some lineages within these haplogroups are only found in Hainan Island; 3) certain sub-differentiation within these haplogroups are observed. Detailed phylogeographic analyses based on mtDNA genomes suggested the initial peopling of Hainan Island was likely to be around 7 - 27 kya (Figure 5 - 6) when Hainan Island was connected with the mainland southern China and/or northern Vietnam [3,9]. The long-standing connection between Hainan Island and the mainland from the LGM to 6 - 7 kya [3,9] could provide the opportunity for some of the dispersals of modern human. Our results are largely in agreement with the time estimates from NRY [4] and are supported by the recent archeological findings [5-8]. However, as mentioned above, the gene pool of Hainan islanders was likely to be affected by the recent immigrants from the mainland. To pin down the recent gene flow and the ancient components in detail, it is necessary to improve the resolution of molecular markers, together with extensive sampling, and even to employ genome-wide autosomal markers, which could be the future direction and would provide more details about the peopling of Hainan Island.

## Conclusions

Combining the fresh data of the mtDNA variation of the 285 Li individuals and those from previous study, we not only help to further understand the mtDNA phylogeny in Hainan Island but also provide deeper insights into the peopling of Hainan Island. Although some genetic differentiations from the populations in the mainland did emerge, in general, the mtDNA phylogeny in Hainan Island was represented as a subset in the context of East Asian and Southeast Asian. The ancestors of the Li people were likely from the populations in mainland southern China, especially in Guangxi. The recent gene flow from the mainland might play important roles in shaping the maternal pool of Hainan islanders. Based on the mtDNA genome sequencing, the phylogeographic analyses of haplogroups M12, M7e, and M7c1* suggested that the related immigration from mainland southern China and Vietnam could be trace back to around 7 - 27 kya, which largely corresponds to the results from NRY and archaeology.

## Methods

### Population samples and DNA extraction

In total, we collected samples from 285 unrelated Li individuals residing in Hainan Island (Figure 2): 86 from Qiongzhong Li and Miao Autonomous County (Li-QZ); 99 from Baoting Li and Miao Autonomous County (Li-BT); and 100 from Ledong Li Autonomous County (Li-LD). All subjects were interviewed to ascertain their ethnic affiliations and to obtain informed consent before blood collection. Comparative mtDNA data from southern China and Vietnam were taken from previous published literature (see Additional file 2). Genomic DNA was extracted from whole blood samples by the standard phenol/chloroform methods.

### MtDNA typing

The mtDNA control region sequences were amplified by the PCR method previously reported [46]. HVS-I (minimum length sequenced was nucleotide positions (np) 16080-16569; maximum length sequenced np 16001-16569) and HVS-II (minimum length sequenced np 1-207; maximum length sequenced np 1-575) were sequenced in all samples as described elsewhere [47]. We performed haplogroup-specific control region motif recognition and (near-) matching search with the published mtDNA data to assign each mtDNA into specific, named haplogroups [46]. Then we selected certain mtDNAs from sequences having similar HVS motifs to genotype the related diagnostic sites in the coding region to confirm their haplogroup status (see Additional file 1). Moreover, 14 whole mtDNA genomes were sequenced following protocols reported elsewhere [48-50]. The sequences generated in this study have been deposited in GenBank database (Accession Nos. HQ156470-HQ156754 for HVS and HQ157971-HQ157984 for mtDNA genome sequences).

Sequences were edited and aligned by Lasergene (DNAStar Inc., Madison, Wisconsin, USA) and mutations were scored relative to the revised Cambridge sequence (rCRS) [51]. For the length variants in the control region, we followed the rules proposed by Bandelt and Parson (2008) [52]. The transition at 16519 and the C-length polymorphisms in regions 16180-16193 and 303-315 were disregarded in the analyses. The classification of the mutations of each mtDNA genomes was performed with mtDNA GeneSyn 1.0 http://www.ipatimup.pt/downloads/mtDNAGeneSyn.zip[53]. To avoid any nomenclature conflicts, we followed the criterion of PhyloTree (http://www.phylotree.org, mtDNA tree Build 10) [54] and the recent updating mtDNA phylogeny in East Asia [28].

### Data analyses

For HVS data, we constructed the median-joining network using Network 4.510 http://www.fluxus-engineering.com/sharenet.htm[55]. The coalescent age of a haplogroup of interest was estimated by statistics ρ ± σ [56,57] and the rate of 18,845 years per transition for control region (16090-16365) [58] was used (Table 2). Principal components analysis (PCA) followed the method developed by Richards et al. with SPSS13.0 software (SPSS) [38]. Analysis of molecular variance (AMOVA) was computed with the package Arlequin 3.11 http://cmpg.unibe.ch/software/arlequin3/[59]. The counter map of spatial frequency was created by using the Kriging algorithm of the Surfer 8.0 package (Golden Software Inc., Golden, Colorado, USA) [60]. To detect the recent gene flow, haplotypes sharing analyses between the Hainan islanders and the populations from the mainland were carried out based on phylogeny [61].

For complete mtDNA sequences, the phylogeny was reconstructed manually and checked by Network 4.510. Six reported sequences were employed for tree reconstructed (Figure 5). To estimate the coalescence time of haplogroup M7c1, additional 13 complete mtDNA genomes from the published literature (Accession Nos. EF153823, EF397561, EU007890, EU597541, AP008755, AP008336, AP008647, AP008886, AP010681, AP010827, HM030514, HM030523, and HM030547) [18,27,28,62-65] were employed but not displayed in the tree. The coalescent age was also estimated by statistics ρ ± σ [56,57]. The recent calibrated rates for the entire mtDNA genome [58] and for only the synonymous mutation [66] were adopted, respectively (Figure 5).

## Authors' contributions

MSP: contributed to the experiment work, data analysis and manuscript writing; JDH: carried out the experiment work and data analysis; HXL: performed the experiment work and commented the manuscript; YPZ: designed the study and prepared the manuscript; and all authors have read and approved the final manuscript.

## Supplementary Material

Additional file 1 **mtDNA control and coding region information of 285 Li individuals from three populations**. The data provide the markers examined in the subjects of the present study. Click here for file

Additional file 2 **Information of comparative populations used in this study**. The information includes ethnic groups, sample sizes, geographic locations, language affinities, and the references for all additional files. Click here for file

Additional file 3 **mtDNA haplogroup frequencies of 50 populations from Hainan Island, mainland southern China, and Vietnam**. The data were used in the PCA and AMOVA. Click here for file

Additional file 4 **Haplotype sharing between Hainan islanders and the populations in the neighboring mainland region**. The haplotype sharing was calculated with the fragment 16090 - 16365 in HVS-I. Click here for file

Additional file 5 **Median-joining network of HVS-I sequences of haplogroups B4b1, B5a, F1a1, R9b, and M7b1**. The information refers sequences from populations in Hainan Island and its neighboring regions in the mainland. Click here for file

Additional file 6 **mtDNA sequences of haplogroup M12**. The information refers 98 sequences of haplogroup M12 used to reconstruct the median-joining network. Click here for file

Additional file 7 **mtDNA sequences of haplogroup M7e**. The information refers 35 sequences of haplogroup M7e used to reconstruct the median-joining network. Click here for file
