# Supplementary material for: Tracing the legacy of the early Hainan Islanders - a perspective from mitochondrial DNA
Source: BMC Evol Biol. 2011 Feb 15;11:46. doi: 10.1186/1471-2148-11-46 (PMC3048540; doi:10.1186/1471-2148-11-46)
Supplement: Additional file 2 — Information of comparative populations used in this study. The information includes ethnic groups, sample sizes, geographic locations, language affinities, and the references for all additional files. [file 1471-2148-11-46-S2.DOC]

**Additional file 2:** Information of comparative populations used in this study.

| **No.** | **Region** | **ID** | **Sample Size** | **Language** | **Population** | **Location** | **References** |
| --- | --- | --- | --- | --- | --- | --- | --- |
| 1 | Guangdong | Han_GZ | 69 | Sinitic | Han | Guangzhou, Guangdong | [1] |
| 2 |  | Han_ZJ | 30 | Sinitic | Han | Zhanjiang, Guangdong | [2] |
| 3 |  | Han_DG | 106 | Sinitic | Han | Dongguan, Guangdong | [3] |
| 4 |  | Hakka | 170 | Sinitic | Hakka | Meizhou, Guangdong | [4] |
| 5 |  | Chaoshan | 102 | Sinitic | Chaoshan | Chanshan, Guangdong | [4] |
| 6 |  | BaPai | 35 | Hmong-Mien | Ba Pai | Liannan, Guangdong | [5] |
| 7 |  | Pou | 34 | Tai-Kadai | Pou | Huaiji, Guangdong | [6] |
| 8 | Hong Kong | HK | 377 | Sinitic | residents of urban Hong Kong with diverse Chinese ancestry | Hong Kong | [7] |
| 9 | Guangxi | BuNu | 19 | Hmong-Mien | Bu Nu | Dahua, Guangxi | [5] |
| 10 |  | HuaTou | 19 | Hmong-Mien | Hua Tou | Fangcheng, Guangxi | [5] |
| 11 |  | Mien_SS | 32 | Hmong-Mien | Mien | Shangsi, Guangxi | [5] |
| 12 |  | Pan | 32 | Hmong-Mien | Pan | Tianlin, Guangxi | [5] |
| 13 |  | TuYao | 41 | Hmong-Mien | Tu Yao | Hezhou, Guangxi | [5] |
| 14 |  | LanTin | 26 | Hmong-Mien | Lan Tin | Tianlin, Guangxi | [5] |
| 15 |  | Lowland | 42 | Hmong-Mien | Lowland | Fuchuang, Guangxi | [5] |
| 16 |  | Wuzhou | 31 | Hmong-Mien | Wuzhou | Fuchuang, Guangxi | [5] |
| 17 |  | Han_TL | 26 | Sinitic | Han | Tianlin, Guangxi | [8] |
| 18 |  | Caolan | 31 | Tai-Kadai | Caolan | Fangcheng, Guangxi | [6] |
| 19 |  | Zhuang_N | 25 | Tai-Kadai | Zhuang | Tianlin, Guangxi | [6] |
| 20 |  | Blue_Gelao | 30 | Tai-Kadai | Blue Gelao | Longlin, Guangxi | [6] |
| 21 |  | Sui | 30 | Tai-Kadai | Sui | Rongshui, Guangxi | [6] |
| 22 |  | Mulam | 66 | Tai-Kadai | Mulam | Luocheng, Guangxi | [6, 9] |
| 23 |  | Maonan | 32 | Tai-Kadai | Maonan | Huanjiang, Guangxi | [6] |
| 24 |  | Palyu | 30 | Austro-Asiatic | Palyu | Longlin, Guangxi | [6] |
| 25 |  | E | 33 | Tai-Kadai | E | Rongshui, Guangxi | [6] |
| 26 |  | Mien_FC | 29 | Hmong-Mien | Mien | Fuchuan, Guangxi | [9] |
| 27 |  | PH_FC | 48 | Sinitic | Pinghua Han | Fuchuan, Guangxi | [9] |
| 28 |  | PH_HZ | 39 | Sinitic | Pinghua Han | Hezhou, Guangxi | [9] |
| 29 |  | Zhuang_HZ | 55 | Tai-Kadai | Zhuang | Hezhou, Guangxi | [9] |
| 30 |  | Laka | 67 | Tai-Kadai | Laka | Jinxiu, Guangxi | [9] |
| 31 |  | PH_LC | 51 | Sinitic | Pinghua Han | Luocheng, Guangxi | [9] |
| 32 |  | Kam | 72 | Tai-Kadai | Kam | Sanjiang, Guangxi | [9] |
| 33 |  | Yerong | 15 | Tai-Kadai | Yerong | Napo, Guangxi | [6] |
| 34 |  | Zhuang_S | 12 | Tai-Kadai | Zhuang | Chongzuo/Shangsi, Guangxi | [6] |
| 35 |  | MuBin | 6 | Hmong-Mien | Mu Bin | Tianlin, Guangxi | [5] |
| 36 |  | XiBan | 11 | Hmong-Mien | Xi Ban | Fangcheng, Guangxi | [5] |
| 37 |  | JX_Pinghua | 13 | Sinitic | Pinghua Han | Jinxiu, Guangxi | [9] |
| 38 |  | Zhuang | 86 | Tai-Kadai | Zhuang | Guangxi | [10] |
| 39 |  | PH_WX | 46 | Sinitic | Pinghua Han | Wuxuan, Guangxi | [9] |
| 40 | Yunnan | Pubiao | 25 | Tai-Kadai | Pubiao | Malipo, Yunnan | [6] |
| 41 |  | Bugan | 32 | Austro-Asiatic | Bugan | Xichou, Yunnan | [6] |
| 42 |  | Buyang | 31 | Tai-Kadai | Buyang | Guangnan, Yunnan | [6] |
| 43 |  | Lachi | 30 | Tai-Kadai | Lachi | Maguan, Yunnan | [6] |
| 44 |  | White_Gelao | 14 | Tai-Kadai | White Gelao | Malipo, Yunnan | [6] |
| 45 |  | KimMun | 40 | Hmong-Mien | Kim Mun | Malipo, Yunnan | [5] |
| 46 | Vietnam | N_Viet | 187 | Austro-Asiatic | Vietnamese | Hanoi, Vietnam | [11] |
| 47 |  | Kinh_HA | 139 | Austro-Asiatic | Kinh | Hanoi, Vietnam | [12] |
| 48 |  | Kinh_HU | 41 | Austro-Asiatic | Kinh | Hue, Vietnam | [6] |
| 49 |  | Bana | 3 | Austro-Asiatic | Bana | Kontum, Vietnam | [6] |
| 50 |  | Chut | 1 | Austro-Asiatic | Chut | Quangbinh, Vietnam | [6] |
| 51 |  | Die | 2 | Austro-Asiatic | Die | Kontum, Vietnam | [6] |
| 52 |  | Hre | 1 | Austro-Asiatic | Hre | Quangngai, Vietnam | [6] |
| 53 |  | Halang | 1 | Austro-Asiatic | Halang | Kontum, Vietnam | [6] |
| 54 |  | Katu | 2 | Austro-Asiatic | Katu | Quangnam, Vietnam | [6] |
| 55 |  | Mnong | 3 | Austro-Asiatic | Mnong | Daklak, Vietnam | [6] |
| 56 |  | Man_Thanth | 2 | Tai-Kadai | Man Thanth | Hatinh, Vietnam | [6] |
| 57 |  | Pacoh | 3 | Austro-Asiatic | Pacoh | Quangtri, Vietnam | [6] |
| 58 |  | Sedang | 1 | Austro-Asiatic | Sedang | Kontum, Vietnam | [6] |
| 59 |  | Trieng | 2 | Austro-Asiatic | Trieng | Kontum, Vietnam | [6] |
| 60 |  | Tay | 4 | Tai-Kadai | Tay | Gialai, Vietnam | [6] |
| 61 | Hainan | Cun | 30 | Tai-Kadai | Cun | Dongfang, Hainan | [6] |
| 62 |  | Danga | 40 | Unclassified | Danga | Lingshui, Hainan | [6] |
| 63 |  | Jiamao | 27 | Tai-Kadai | Jiamao | Baoting, Hainan | [6] |
| 64 |  | Lingao | 31 | Tai-Kadai | Lingao | Lingao, Hainan | [6] |
| 65 |  | Li_TZ | 34 | Tai-Kadai | Li | Tongza (Wuzhishan), Hainan | [6] |
| 66 |  | Li_BT | 99 | Tai-Kadai | Li | Qiongzhong, Hainan | This Study |
| 67 |  | Li_LD | 100 | Tai-Kadai | Li | Ledong, Hainan | This Study |
| 68 |  | Li_QZ | 86 | Tai-Kadai | Li | Baoting, Hainan | This Study |

**References**

1. Kivisild T, Tolk HV, Parik J, Wang YM, Papiha SS, Bandelt HJ, Villems R: **The emerging limbs and twigs of the East Asian mtDNA tree.** *Mol Biol Evol* 2002, **19:**1737-1751.

2. Yao YG, Kong QP, Bandelt HJ, Kivisild T, Zhang YP: **Phylogeographic differentiation of mitochondrial DNA in Han Chinese.** *Am J Hum Genet* 2002, **70:**635-651.

3. Chen F, Wang SY, Zhang RZ, Hu YH, Gao GF, Liu YH, Kong QP: **Analysis of mitochondrial DNA polymorphisms in Guangdong Han Chinese.** *Forensic Sci Int Genet* 2008, **2:**150-153.

4. Wang WZ, Wang CY, Cheng YT, Xu AL, Zhu CL, Wu SF, Kong QP, Zhang YP: **Tracing the origins of Hakka and Chaoshanese by mitochondrial DNA analysis.** *Am J Phys Anthropol* 2010, **141:**124-130.

5. Wen B, Li H, Gao S, Mao X, Gao Y, Li F, Zhang F, He Y, Dong Y, Zhang Y, et al: **Genetic structure of Hmong-Mien speaking populations in East Asia as revealed by mtDNA lineages.** *Mol Biol Evol* 2005, **22:**725-734.

6. Li H, Cai X, Winograd-Cort ER, Wen B, Cheng X, Qin Z, Liu W, Liu Y, Pan S, Qian J, et al: **Mitochondrial DNA diversity and population differentiation in Southern East Asia.** *Am J Phys Anthropol* 2007, **134:**481-488.

7. Irwin JA, Saunier JL, Beh P, Strouss KM, Paintner CD, Parsons TJ: **Mitochondrial DNA control region variation in a population sample from Hong Kong, China.** *Forensic Sci Int Genet* 2009, **3:**e119-125.

8. Wen B, Li H, Lu D, Song X, Zhang F, He Y, Li F, Gao Y, Mao X, Zhang L, et al: **Genetic evidence supports demic diffusion of Han culture.** *Nature* 2004, **431:**302-305.

9. Gan RJ, Pan SL, Mustavich LF, Qin ZD, Cai XY, Qian J, Liu CW, Peng JH, Li SL, Xu JS, et al: **Pinghua population as an exception of Han Chinese's coherent genetic structure.** *J Hum Genet* 2008, **53:**303-313.

10. Yao YG, Nie L, Harpending H, Fu YX, Yuan ZG, Zhang YP: **Genetic relationship of Chinese ethnic populations revealed by mtDNA sequence diversity.** *Am J Phys Anthropol* 2002, **118:**63-76.

11. Irwin JA, Saunier JL, Strouss KM, Diegoli TM, Sturk KA, O'Callaghan JE, Paintner CD, Hohoff C, Brinkmann B, Parsons TJ: **Mitochondrial control region sequences from a Vietnamese population sample.** *Int J Legal Med* 2008, **122:**257-259.

12. Peng MS, Quang HH, Dang KP, Trieu AV, Wang HW, Yao YG, Kong QP, Zhang YP: **Tracing the Austronesian Footprint in Mainland Southeast Asia: A Perspective from Mitochondrial DNA.** *Mol Biol Evol*, **27:**2417-2430.

13. Hill C, Soares P, Mormina M, Macaulay V, Meehan W, Blackburn J, Clarke D, Raja JM, Ismail P, Bulbeck D, et al: **Phylogeography and ethnogenesis of aboriginal Southeast Asians.** *Mol Biol Evol* 2006, **23:**2480-2491.

14. Wong HY, Tang JS, Budowle B, Allard MW, Syn CK, Tan-Siew WF, Chow ST: **Sequence polymorphism of the mitochondrial DNA hypervariable regions I and II in 205 Singapore Malays.** *Leg Med (Tokyo)* 2007, **9:**33-37.

15. Tajima A, Hayami M, Tokunaga K, Juji T, Matsuo M, Marzuki S, Omoto K, Horai S: **Genetic origins of the Ainu inferred from combined DNA analyses of maternal and paternal lineages.** *J Hum Genet* 2004, **49:**187-193.

16. Hill C, Soares P, Mormina M, Macaulay V, Clarke D, Blumbach PB, Vizuete-Forster M, Forster P, Bulbeck D, Oppenheimer S, Richards M: **A mitochondrial stratigraphy for island southeast Asia.** *Am J Hum Genet* 2007, **80:**29-43.

17. Maruyama S, Nohira-Koike C, Minaguchi K, Nambiar P: **MtDNA control region sequence polymorphisms and phylogenetic analysis of Malay population living in or around Kuala Lumpur in Malaysia.** *Int J Legal Med* 2009, **16:**16.

18. Cordaux R, Saha N, Bentley GR, Aunger R, Sirajuddin SM, Stoneking M: **Mitochondrial DNA analysis reveals diverse histories of tribal populations from India.** *Eur J Hum Genet* 2003, **11:**253-264.

19. Chandrasekar A, Kumar S, Sreenath J, Sarkar BN, Urade BP, Mallick S, Bandopadhyay SS, Barua P, Barik SS, Basu D, et al: **Updating phylogeny of mitochondrial DNA macrohaplogroup m in India: dispersal of modern human in South Asian corridor.** *PLoS ONE* 2009, **4:**e7447.

20. Trejaut JA, Kivisild T, Loo JH, Lee CL, He CL, Hsu CJ, Li ZY, Lin M: **Traces of archaic mitochondrial lineages persist in Austronesian-speaking Formosan populations.** *PLoS Biol* 2005, **3:**1362-1372.

21. Family Tree DNA - Genealogy by Genetics, Ltd. http://www.familytreedna.com/

22. Horai S, Murayama K, Hayasaka K, Matsubayashi S, Hattori Y, Fucharoen G, Harihara S, Park KS, Omoto K, Pan IH: **mtDNA polymorphism in East Asian populations, with special reference to the peopling of Japan.** *Am J Hum Genet* 1996, **59:**579-590.

23. Jin HJ, Tyler-Smith C, Kim W: **The Peopling of Korea Revealed by Analyses of Mitochondrial DNA and Y-Chromosomal Markers.** *PLoS ONE* 2009, **4:**e4210.

24. Lertrit P, Poolsuwan S, Thosarat R, Sanpachudayan T, Boonyarit H, Chinpaisal C, Suktitipat B: **Genetic history of Southeast Asian populations as revealed by ancient and modern human mitochondrial DNA analysis.** *Am J Phys Anthropol* 2008, **137:**425-440.

25. Fucharoen G, Fucharoen S, Horai S: **Mitochondrial DNA polymorphisms in Thailand.** *J Hum Genet* 2001, **46:**115-125.

26. Oota H, Settheetham-Ishida W, Tiwawech D, Ishida T, Stoneking M: **Human mtDNA and Y-chromosome variation is correlated with matrilocal versus patrilocal residence.** *Nat Genet* 2001, **29:**20-21.

27. Kong QP, Bandelt HJ, Sun C, Yao YG, Salas A, Achilli A, Wang CY, Zhong L, Zhu CL, Wu SF, et al: **Updating the East Asian mtDNA phylogeny: a prerequisite for the identification of pathogenic mutations.** *Hum Mol Genet* 2006, **15:**2076-2086.

28. Wen B, Xie XH, Gao S, Li H, Shi H, Song XF, Qian TZ, Xiao CJ, Jin JZ, Su B, et al: **Analyses of genetic structure of Tibeto-Burman populations reveals sex-biased admixture in southern Tibeto-Burmans.** *Am J Hum Genet* 2004, **74:**856-865.

29. Qian YP, Chu ZT, Dai Q, Wei CD, Chu JY, Tajima A, Horai S: **Mitochondrial DNA polymorphisms in Yunnan nationalities in China.** *J Hum Genet* 2001, **46:**211-220.

30. Nur Haslindawaty AR, Panneerchelvam S, Edinur HA, Norazmi MN, Zafarina Z: **Sequence polymorphisms of mtDNA HV1, HV2, and HV3 regions in the Malay population of Peninsular Malaysia.** *Int J Legal Med* 2010, **124:**415-426.

31. Oota H, Kitano T, Jin F, Yuasa I, Wang L, Ueda S, Saitou N, Stoneking M: **Extreme mtDNA homogeneity in continental Asian populations.** *Am J Phys Anthropol* 2002, **118:**146-153.

32. Yao YG, Zhang YP: **Phylogeographic analysis of mtDNA variation in four ethnic populations from Yunnan Province: new data and a reappraisal.** *J Hum Genet* 2002, **47:**311-318.

33. Derenko M, Malyarchuk B, Grzybowski T, Denisova G, Dambueva I, Perkova M, Dorzhu C, Luzina F, Lee HK, Vanecek T, et al: **Phylogeographic analysis of mitochondrial DNA in northern Asian Populations.** *Am J Hum Genet* 2007, **81:**1025-1041.

34. Kong QP, Sun C, Wang HW, Zhao M, Wang WZ, Zhong L, Hao XD, Pan H, Wang SY, Cheng YT, et al: **Large-scale mtDNA screening reveals a surprising matrilineal complexity in East Asia and its implications to the peopling of the region.** *Mol Biol Evol* 2011, **28:**513-522.
