# Supplementary figures and images for: Tracing the legacy of the early Hainan Islanders - a perspective from mitochondrial DNA
Source: BMC Evol Biol. 2011 Feb 15;11:46. doi: 10.1186/1471-2148-11-46 (PMC3048540; doi:10.1186/1471-2148-11-46)

Additional file 5

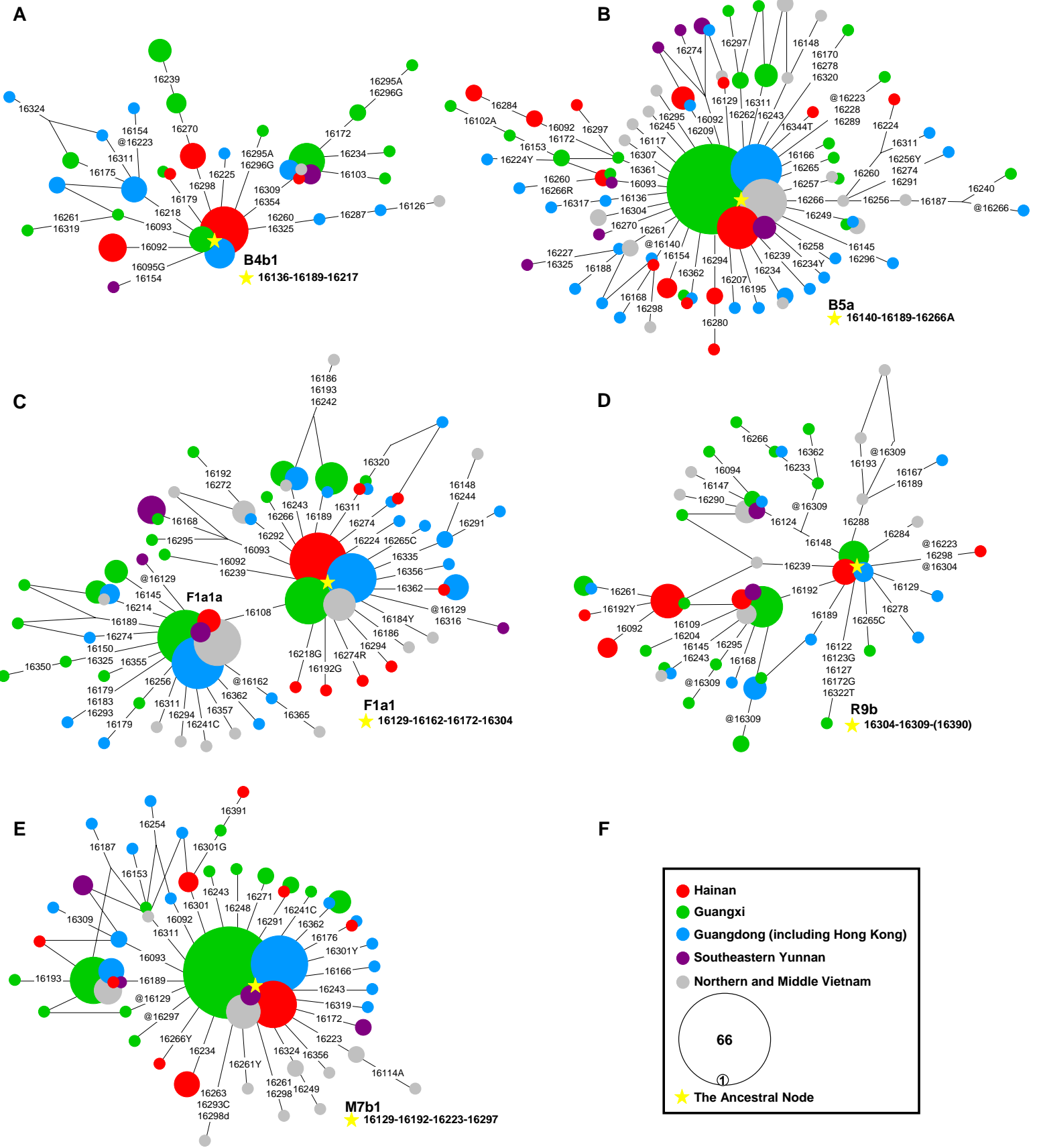

Supplement: Additional file 5 — Median-joining network of HVS-I sequences of haplogroups B4b1, B5a, F1a1, R9b, and M7b1. The information refers sequences from populations in Hainan Island and its neighboring regions in the mainland. [file 1471-2148-11-46-S5.PDF]
